# Optimization of Submerged Culture Parameters of the Aphid Pathogenic Fungus *Fusarium equiseti* Based on Sporulation and Mycelial Biomass

**DOI:** 10.3390/microorganisms11010190

**Published:** 2023-01-12

**Authors:** Xueyi Zhao, Junfa Chai, Fang Wang, Yanxia Jia

**Affiliations:** 1 College of Agriculture, Ningxia University, Yinchuan 750021, China; 2 Institute of Plant Protection, Ningxia Academy of Agriculture and Forestry Sciences, Yinchuan 750002, China

**Keywords:** *Fusarium equiseti*, optimal submerged culture conditions, response surface methodology, box–behnken design, *Myzus persicae*

## Abstract

*Fusarium equiseti* (JMF-01), as an entomopathogenic fungus, can effectively control agricultural pests and has the potential to be a biocontrol agent. To promote mycelial growth and sporulation, we investigated the optimal submerged culture conditions for *F. equiseti*. In this study, we used the single-factor method and Box–Behnken design and determined the virulence of the submerged culture against *Myzus persicae* after optimization. As a result, the highly significant factors affecting the spore concentration of strain JMF-01 were the primary inoculum density and the initial pH, and the highly significant factor affecting the mycelial biomass was the medium-to-flask ratio. The highest mycelial biomass value was 0.35 g when the incubation time was 5.68 days, the initial pH was 5.11, the medium-to-flask ratio was 0.43, and 1 mL of the primary inoculum with spore density of 0.97 × 10^7^ conidia/mL was added. When the incubation time was 6.32 days, the initial pH was 4.46, the medium-to-flask ratio was 0.35, the primary inoculum density was 1.32 × 10^7^ conidia/mL of 1 mL, and the highest spore concentration of 6.49 × 10^8^ blastospores/mL was obtained. Compared with the unoptimized medium conditions, the optimized submerged culture had the highest mycelial biomass and spore concentration, which were 3.46 and 2.06 times higher, respectively. The optimized submerged culture was highly pathogenic toward *M. persicae*, reaching a 95% mortality rate. Our results provide optimal submerged culture conditions for *F. equiseti* and lay the basis for later research to expand production for pest control.

## 1. Introduction

Entomopathogenic fungi (EPF) are known to be beneficial natural enemies of insect pests [1]. Ascomycota and Entomophtoromycota include most species of entomopathogenic fungi [2]. EPF are mainly used in the environment to control pest populations [3], and the increased need for environmentally friendly pest management technologies has resulted in an increase in the use of organic biocontrollers such as microorganisms, particularly EPF [4].

Due to the fact of their prevalence in the soil and frequent connection with plant roots as saprophytes, *Fusarium* species are commonly referred to as soil-borne fungi [5]. *Fusarium* species are commonly reported as plant pathogens that cause serious diseases in a variety of plant species [6]. However, it has been demonstrated that a number of *Fusarium* species have moderate to severe levels of insect pathogenicity, primarily against homopterous and dipterous insects [7,8]. In terms of EPF, *Fusarium* species represent an important member and have the potential to be effective agents against insects [9]. The use of entomopathogenic *Fusarium* species for the biological management of insects is advantageous because some *Fusarium* isolates cause high mortalities in their insect host, do not infect the crop plant, and can also be easily propagated in the laboratory [10,11]. Several studies report the insect pathogenicity of *Fusarium* spp., either in the laboratory or on-field natural mycoses, such as *Fusarium avenaceum* and *Fusarium verticillioides* are pathogenic to *Sitophilus oryzae* and *Tropidacris collaris*, respectively [12]. *Fusarium equiseti* was isolated from *Bemisia tabaci* and resulted in a significant rate of mortality in *B. tabaci* nymphs [13]. *Fusarium incarnatum-equiseti* species complex (FIESC) and *F. equiseti* were reported to be insect pathogenic fungi that were isolated from insects and soil, which can be used to control pests [14,15,16,17,18]. FIESC isolates and extracts of *Ricinus communis* and *Poincianella pyramidalis* were successfully employed by da Silva Santos et al. [19] against *Dactylopius opuntiae*. Beauvericin, a cyclodepsipeptide toxin discovered from *Fusarium* by Gupta et al. [20], is toxic to the Colorado potato beetle. In addition, *F. equiseti* can also be used for pest control by producing insecticidal toxins [21,22].

*Myzus persicae* is one of the most diverse and polyphagous agricultural pests, which infests crops directly by draining the phloem sap or spreading viral infections from the seedling stage to harvest stage [23]. On the one hand, *M. persicae* is exceedingly difficult to manage solely with pesticides due to increased insecticide resistance and rapid increases in population size [24]. On the other hand, concerns regarding the excessive use of chemical pesticides make it necessary to seek safe and effective alternatives. Thus, mycoinsecticides are attracting considerable attention as environmentally friendly pest management agents [25].

Two methods of fungal growth are solid and submerged cultures. Solid culture is more favorable for sporulation because most fungi sporulate on solid media, which is easy to achieve in the laboratory, but this method is subject to many technical and economic limitations [26]. Submerged culture overcomes these restrictions by providing careful control of the initial pH, dissolved oxygen (DO) concentration, and inoculum volume to shorten incubation time, lessen labor, provide an optimal growth environment for strain growth, and reduce the risk of contamination [27,28]. Most EPF take longer to produce conidia on solid substrates and only a few days to produce blastospores on submerged media [29,30], and some studies have concluded that blastospores are more virulent than conidia [30,31,32]. However, subtle variations in host-pathogen interactions can lead to significant differences in pathogenicity [33]. Conidia of EPF often infect their hosts through a unique pathogenicity process that involves the successful adhesion, germination, differentiation, and penetration of fungal hyphae [34,35]. The blastospore, which differs from conidia, can produce copious mucilage, adhere readily to the cuticle surface, and invade through the gut following ingestion [31]. Typically, spore adhesion determines how successfully an infection spreads [7,36]. *Fusarium* species usually produce two types of conidia, namely macroconidia and microconidia [10,37]. Microconidia may be phialospores or blastospores [38,39]. This is due to the fact that the optimal culture conditions and medium composition vary depending on the species and occasionally even among strains of some species [40]. Therefore, we hypothesized that the production of blastospore and mycelia would reach its optimum value under submerged culture and be pathogenic to *M. persicae* during our tests.

Response surface methodology (RSM) is used to find the ideal combination of conditions and components that influence the biological process by measuring the response of each variable and assessing the interactions of groups of controlled factors in an experimental set [41]. Compared with the traditional techniques, RSM can not only overcome the limitations of economy and time, but also evaluate many parameters and their interactions through fewer experiments [42]. A mathematical modeling and statistical method called the Box–Behnken design (BBD) of RSM aids in optimizing conditions [43]. Previous studies used a RSM to optimize biological processes [44,45,46,47,48], BBD also has been applied in the optimization of the yield of EPF in several studies [40,49,50]. However, it has not been applied to entomopathogenic *Fusarium* spp. Our preliminary results showed that a *Fusarium* strain, JMF-01, isolated from the cadavers of *M. persicae* and identified as *Fusarium equiseti*, has promising prosperities as a biological pesticide for *M. persicae* [51]. Therefore, our study used the two stepwise enhancement strategies [52] to optimize the four independent culture conditions related to spore production and mycelial biomass of strain JMF-01. First, the single-factor method was utilized to evaluate the factors’ optimum values, and then the level of signed factors was optimized by the BBD method. This provides optimal submerged culture conditions for strain JMF-01 and lays the foundation for the subsequent application of *F. equiseti* as a biocontrol agent in agriculture.

## 2. Materials and Methods

### 2.1. Organism, Inoculum Preparation, and Culture Conditions

Strain JMF-01 was previously isolated from dead *M. persicae* in Helan County (Ningxia, China) in 2020. The isolation procedure was as follows; soaking the dead individuals of *M. persicae* in a 75% ethanol solution for 10 s, then rinsing them three times with sterile water. Cadavers were then dried on filter paper and put on PDA medium with 0.03% chloramphenicol at (25 ± 1) °C for 3 days [51]. According to Topuz et al. [3], the fresh fungal colony was picked from the edge once spore formation was observed and subcultured on freshly prepared PDA five times to obtain a purified culture. Strain JMF-01 was identified as *Fusarium equiseti* through morphological and molecular techniques [51]. In addition, the strain JMF-01 was inoculated in *M. persicae* to verify pathogenicity. At 4 °C, potato dextrose agar (PDA) plates were used to retain the purified fungus.

A spore suspension of 1 × 10^7^ conidia/mL was prepared in sterile distilled water and used as a source of inoculums.

### 2.2. Experimental Design

#### 2.2.1. Preliminary Experiments: Screening of the Basic Medium

The optimum medium for the growth of strain JMF-01 was screened out of five liquid media: potato dextrose liquid medium, potato sucrose liquid medium, Sabouraud Dextrose Agar with a yeast extract liquid medium, Czapek–Dox liquid medium, and glucose yeast extract liquid medium (Table 1). Each assay was repeated three times.

#### 2.2.2. Single Factor Experimental Design

The single factor test was conducted with the incubation time, the primary inoculum density, the medium-to-flask ratio, and the initial pH value as the factors, and the optimum liquid medium was selected through preliminary experiments as the base medium for each group of experiments. Each treatment was replicated three times. After incubation for 2 days, 2 mL was sampled each day until the 8th day to compare the spore concentration and mycelial biomass of strain JMF-01 under different conditions.

##### Incubation Time

Standard inoculum (1 × 10^7^ conidia/mL, 1 mL) was inoculated in each 150 mL Erlenmeyer flask containing 50 mL of potato sucrose liquid medium (pH 7). Each inoculated Erlenmeyer flask was placed on a shaker (150 rpm/min) at 28 °C for 8 days. After 4, 5, 6, 7, and 8 days of incubation, 2 mL was sampled to compare the spore concentration and mycelial biomass of strain JMF-01 under five sets of time conditions.

##### Initial pH

To investigate the effect of the initial pH on the growth of the strain JMF-01, the initial pH values were adjusted before sterilization using HCl (GR) or NaOH (AR) to 4.0, 5.0, 6.0, 7.0, and 8.0. One milliliter of spore suspension (1 × 10^7^ conidia/mL) was inoculated for each treatment and placed on a shaker (150 rpm/min) at 28 °C for 8 days.

##### Medium-to-Flask Ratio

The effect on the growth of the strain JMF-01 was investigated by setting the medium-to-flask ratio (medium volume-to-flask volume ratio) to one-third, one-quarter, one-fifth, and one-sixth. The inoculated Erlenmeyer flask was placed on a shaker (150 rpm/min) at 28 °C for 6 days.

##### Primary Inoculum Density

To study the effect of the primary inoculum density on the spore concentration and mycelial biomass of strain JMF-01, five groups of inoculum levels were set at 0.25, 0.5, 1, 1.5, and 2 (10^7^ conidia/mL), and one milliliter of spore suspension was added to each Erlenmeyer flask. At 28 °C (150 rpm/min), the inoculated Erlenmeyer flasks were set on a shaker.

#### 2.2.3. Assessment of Sporulation and Mycelial Production

The liquid medium at the end of incubation was centrifuged (10,000 r/min for 20 min) or filtered by gauze, and then the mycelium was placed in a drying oven and dried at 85 °C to a constant weight, and its mycelium dry weight was recorded.

The spore concentration was measured using a hemocytometer plate [53].

#### 2.2.4. Box–Behnken Design

The next step was to use the findings of the single-factor experiment to guide the design of each factor and level in a BBD. We used the optimal liquid medium selected through preliminary experiments as the base medium. Response surface optimization was performed with the production of JMF-01 spores and mycelia as the response values, and a four-factor, three-level response analysis was conducted to optimize the four factors affecting the strain JMF-01 submerged culture. The experimental design’s ranges and levels are displayed in Table 2.

The method of multiple regression was then used to fit the data to a second-order polynomial equation. This led to the development of an empirical model that connected the measured response to the experiment’s independent factors. The model equation for a four-factor system is as follows:

$$Y=b_{0}+b_{1}X_{1}+b_{2}X_{2}+b_{3}X_{3}+b_{11}X_{1}^{2}+b_{22}X_{2}^{2}+b_{33}X_{3}^{2}+b_{12}X_{1}X_{2}+b_{13}X_{1}X_{3}+b_{23}X_{2}X_{3}$$

where *Y* stands for the predicted response; *b*_0_ is the mean effect; *b*_1_, *b*_2_, and *b*_3_ are the linear coefficients; *b*_11_, *b*_22_, and *b*_33_ are the squared coefficients; *b*_12_, *b*_13_, and *b*_23_ are the interaction coefficients.

The coefficient of determination (R^2^) determines the polynomial model equation’s goodness of fit. We tested the adequacy of the model using the F-value test and coefficient of determination (R^2^) test. The possibility of improving mycelia and spore production was analyzed using response surface contour plots.

#### 2.2.5. Verification Test

Three validation tests were performed under the optimal submerged culture conditions obtained using Design-Expert 12 software, and the corresponding response values were averaged from the results of the three tests and compared with the predicted response values of the regression model. Unoptimized submerged culture was obtained by initial submerged culture conditions, which was in each 150 mL flask containing 50 mL of the base medium (pH 7) and primary inoculum density was 1 mL (1 × 10^7^ conidia/mL) for 8 days.

### 2.3. Pathogenicity Test

The pathogenicity of the submerged culture of strain JMF-01 under submerged culture against *M. persicae* was evaluated in the laboratory. *Brassica oleracea* leaves were trimmed, and the adult aphids were removed from a colony that was kept in a green-house. Approximately 30 wingless adults were retained on each *Brassica oleracea* leaf disc and sprayed with optimized submerged culture [54]. Optimized submerged culture was screened through preliminary experiments and BBD. After being sprayed with submerged culture, the leaf discs were dried at room temperature and placed in Petri dishes (d = 9 cm) lined with filter paper, wrapped with moist cotton to cover the petiole, sealed with cling film, and tied, allowing for holes to maintain the air circulation. Sterile water was treated as a control. The treatments were incubated in a light incubator at 25 ± 1 °C, RH (80 ± 5) %, and a photoperiod L:D = 14:10 h. Each treatment was replicated three times. The number of dead peach aphids was counted every 24 h, and the peach aphids were observed under a microscope for staining; deaths were recorded by lightly touching the insects with a fine brush. The total observation period was 7 days.

### 2.4. Statistical Analysis

The summary statistics are presented as the mean ± standard error of the mean (SEM) since each experiment was done three times independently. The one-way ANOVA with Tukey HSD test was used to examine the data of the single-factor tests and the verification test. Significant differences between sample mean values were defined as those with *p* < 0.05. Design-Expert software (version 12.0.0, Stat-Ease Inc., Minneapolis, MN, USA) was utilized to evaluate data from the BBD. The survival of *M. persicae* was evaluated using a Kaplan-Meier analysis followed by a log-rank test (*p* < 0.001).

## 3. Results

### 3.1. Single-Factor Experiments

#### 3.1.1. The Effects of the Basic Submerged Medium and Incubation Time on Spore Concentration and Mycelial Biomass

After eight days of incubation treatment in the five liquid media, the spore concentration and mycelial biomass of strain JMF-01 cultured in potato sucrose liquid media were 3.15 × 10^8^ blastospores/mL and 0.10 g/mL, respectively, which were significantly higher than for the other media (Tukey’s test, *p* < 0.05). Therefore, potato sucrose liquid medium was screened as the base medium for each group of experiments.

**Figure 1:**
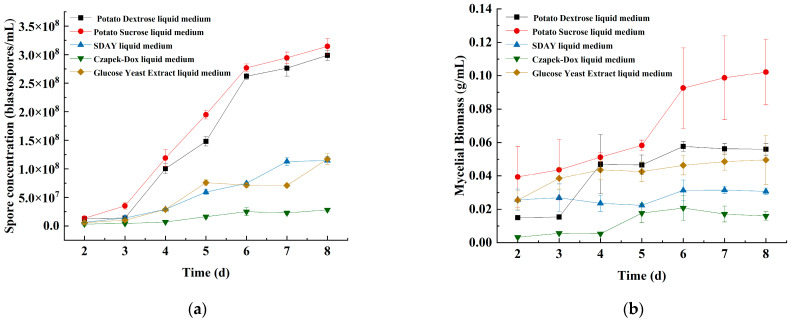
Effects of different medium and incubation time on the (**a**) spore concentration and (**b**) mycelial biomass of strain JMF-01. The data in the figure are the mean ± SEM. The SEM of three repetitions is shown in bars.

After 2 days of incubation, the spore concentration and mycelial biomass showed a rapid growth trend with the incubation time until the 6th day. After 6 days of incubation, the spore concentration grew slowly, while the mycelial biomass decreased slightly. Thus, the optimal incubation time was 6–8 days (Figure 1, Tables S1 and S2).

#### 3.1.2. Effects of the Initial pH on Spore Concentration and Mycelial Biomass

**Figure 2:**
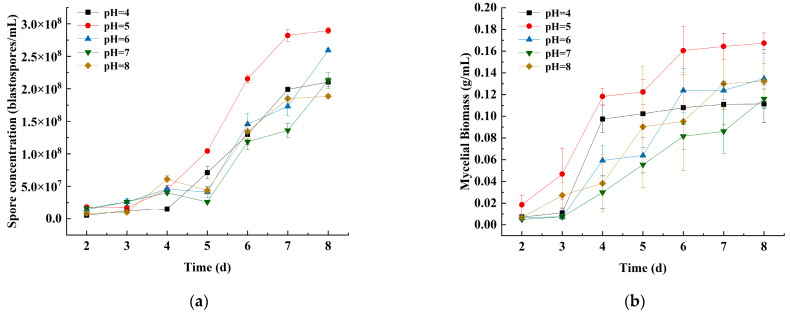
Effects of different initial pH on the (**a**) spore concentration and (**b**) mycelial biomass of strain JMF-01. The data in the figure are the mean ± SEM. The SEM of three repetitions is shown in bars.

The initial pH of the submerged culture had basically the same trend as the spore concentration and mycelial biomass of strain JMF-01. At an initial pH of 4–8, strain JMF-01 could grow, but too strong acidity and alkalinity were not conducive to the growth of strain JMF-01 (Figure 2, Tables S1 and S2). At an initial pH of 5, the spore concentration and mycelial biomass of strain JMF-01 reached the maximum value of 2.90 × 10^8^ blastospores/mL and 0.17 g/mL, respectively (Tukey’s test, *p* < 0.05, compared to others).

#### 3.1.3. Effects of the Primary Inoculum Density on Spore Concentration and Mycelial Biomass

**Figure 3:**
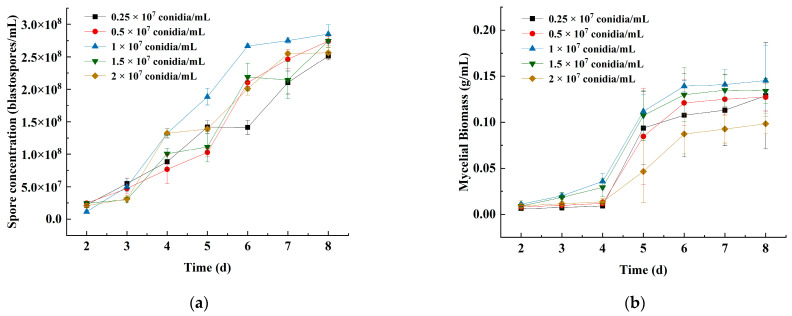
Effects of different primary inoculum density on the (**a**) spore concentration and (**b**) mycelial biomass of strain JMF-01. The data in the figure are the mean ± SEM. The SEM of three repetitions is shown in bars.

The spore concentration and mycelial biomass of strain JMF-01 were highest at an inoculum level of 1 × 10^7^ conidia/mL after 6 days with 2.85 × 10^8^ blastospores/mL (Tukey’s test, *p* < 0.05, compared to other primary inoculum densities) and 0.15 g/mL (Tukey’s test, *p* = 0.73), respectively. However, the spore concentration and mycelial biomass of strain JMF-01 were lower at inoculum levels of 0.25 × 10^7^ conidia/mL and 2 × 10^7^ conidia/mL, indicating that higher or lower inoculum levels were not favorable to the submerged culture of the strain (Figure 3, Tables S1 and S2).

#### 3.1.4. Effects of the Medium-to-Flask Ratio on Spore Concentration and Mycelial Biomass

**Figure 4:**
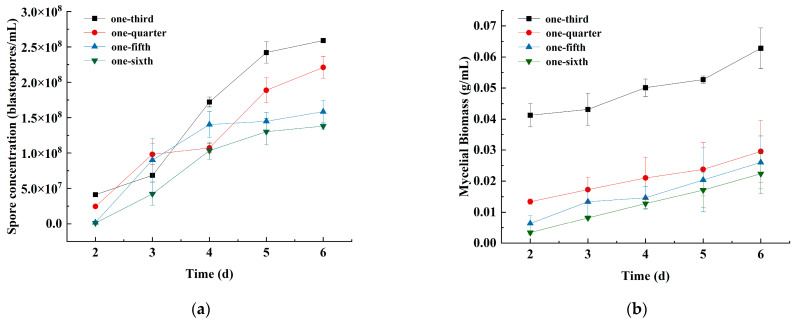
Effects of different medium-to-flask ratio on the (**a**) spore concentration and (**b**) mycelial biomass of strain JMF-01. The data in the figure are the mean ± SEM. The SEM of three repetitions is shown in bars.

The growth curve of strain JMF-01 increased with the increase in the medium-to-flask ratio and reached the highest when the medium-to-flask ratio was one-third (Figure 4, Tables S1 and S2). The spore concentration and mycelial biomass were 2.59 × 10^8^ blastospores/mL and 0.063 g/mL, respectively (Tukey’s test, *p* < 0.05, compared to other treatments). When the medium-to-flask ratio was one-sixth, the spore concentration and mycelial biomass were the lowest (Tukey’s test, *p* < 0.05), indicating that a too low medium-to-flask ratio is not conducive to the growth of strain JMF-01.

The findings revealed that the spore concentration and mycelial biomass of the strain both reached their maximums when the incubation time was 6 days, the initial pH value was 5, the medium-to-flask ratio was one-third, and the primary inoculum density was 1 × 10^7^ conidia/mL.

### 3.2. Box–Behnken Design

The optimal values for the four variables, incubation time, initial pH, medium-to-flask ratio, and primary inoculum density, were chosen as the center level based on the findings of the single factor studies, and the spore concentration and mycelial biomass of strain JMF-01 were used as the response values. The response surface optimization of the four factors and three levels was carried out using Design Expert 12, and the central point experiment was repeated five times for a total of 29 experiments. The coding of each factor is shown in Table 2, and the experimental design and results are shown in Table 3. Multiple regression fitting was performed on the experimental results in Table 3:

$$\begin{array}{l} \text{YS (Spore concentration)}=6.06+0.0799A-0.6576B-0.0361C+0.7542D-0.4437\mathrm{AB}\\ +0.2521\mathrm{AC}+0.4896\mathrm{AD}-0.5292\mathrm{BC}-0.0833\mathrm{BD}-0.2396\mathrm{CD}-1.05A^{2}-0.8847B^{2}-\\ 0.4544C^{2}-0.7190D^{2} \end{array}$$

$$\begin{array}{l} \text{YM (Mycelial biomass)}=0.3053-0.0175A+0.0013B+0.0851C+0.0036D+0.0176\mathrm{AB}\\ +0.0009\mathrm{AC}-0.0059\mathrm{AD}+0.0110\mathrm{BC}+0.0062\mathrm{BD}-0.0095\mathrm{CD}-0.0224A^{2}-0.0287B^{2}-\\ 0.0405C^{2}-0.0251D^{2} \end{array}$$

The analysis of variance for the spore concentration of strain JMF-01 (Table 4) showed that the fitted model was highly significant (*p* < 0.0001), and the out-of-fit term (*p* = 0.4028) was greater than 0.1. The out-of-fit test was not significant, indicating that the model was built to cover all of the data. The difference between the coefficient of determination of the regression model (R^2^ = 0.9355) and the corrected coefficient of determination (adjusted R^2^ = 0.8709) was less than 0.2, indicating that the equation fit well with the experimental results [55]. The significance test of the coefficients in the regression equation showed that B, D, A^2^, B^2^, C^2^, and D^2^ were highly significant (*p* < 0.01), AB, AD, and BC were significant (*p* < 0.05), and the rest were insignificant. The analysis of the F-value showed that the main and secondary factors affecting the spore concentration of strain JMF-01 were primary inoculum density (D) > initial pH (B) > incubation time (A) > medium-to-flask ratio (C).

By analyzing the regression equation for the mycelial biomass of strain JMF-01 (Table 5), the model was found to be highly significant (*p* < 0.0001), and the difference in the out-of-fit term was not significant (0.3428), indicating that the model was reliable. The coefficient of determination of the model, R^2^ = 0.9450, was similar to the corrected coefficient of determination, adjusted R^2^ = 0.8900, indicating that the predicted values of mycelial biomass in the model were highly correlated with the experimental values. In the model, C, B^2^, C^2^, and D^2^ were highly significant, A and A^2^ were significant, and the rest were not significant. The analysis of the F-value showed that the main and secondary factors affecting the mycelial biomass of strain JMF-01 were medium-to-flask ratio (C) > incubation time (A) > primary inoculum density (D) > initial pH (B).

### 3.3. 3D Response Surfaces and 2D Contour Analysis

**Figure 5:**
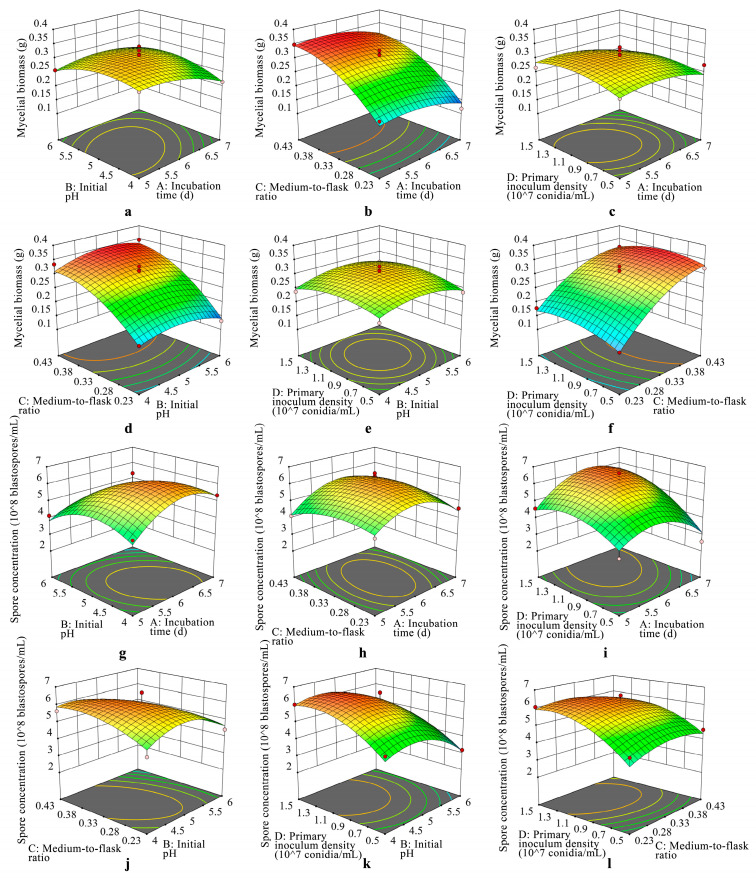
Response surface plots of the mycelial biomass and spore concentration of strain JMF-01. In the figure, (**a**–**f**) are the response surface diagrams of each factor on the mycelial biomass of strain JMF-01; and (**g**–**l**) are the effects on spore concentration.

The response surface and contour plots were drawn by the software Design Expert 12, which could directly reflect the effects of various submerged culture conditions on the spore concentration and mycelial biomass of strain JMF-01 (Figure 5). The more the research factors influence the response value, the steeper the three-dimensional map is in the response surface graphic. When the contour shape is oval, the interaction between factors is significant, and when the contour shape is round, the interaction is not significant. For the contour line, the farther the distance between the center and the surrounding contour line, the greater the slope of the three-dimensional surface map, and the more significant the impact [56].

The effects of the incubation time (A) and the initial pH (B) on the mycelial biomass (Figure 5a) and spore concentration (Figure 5g) of strain JMF-01 were investigated when the medium-to-flask ratio (C) and the primary inoculum density (D) were constant. The mycelial biomass reached its maximum in the interval of an incubation time of 5–6 days and an initial pH of 4.5–5.5, while the spore concentration of strain JMF-01 reached its maximum in the interval of an incubation time of 5.5–6.5 days and an initial pH of 4–5. The spore concentration and mycelial biomass both showed a tendency of growing and then declining with the increase in initial pH when the incubation period was constant, and the spore concentration and mycelial biomass likewise showed this trend when the initial pH was constant. The contours of the spore concentration and mycelial biomass of strain JMF-01 were elliptical, and the response surfaces were parabolic and opened downward, indicating that the incubation time and initial pH had significant effects on the spore concentration and mycelial biomass of the strain.

The effects of the incubation time (A) and the medium-to-flask ratio (C) on the mycelial biomass (Figure 5b) and spore concentration (Figure 5h) of strain JMF-01 were investigated when the initial pH (B) and the primary inoculum density (D) were constant. The spore concentration of strain JMF-01 had the highest value at an incubation time of 5.5–6.5 days and a medium-to-flask ratio of 0.28–0.38. And the contour line was elliptical, indicating that the interaction between incubation time and medium-to-flask ratio was more significant. When the incubation time was constant, the mycelial biomass showed an increasing trend with the increase in the medium-to-flask ratio. The response surface curve had a large slope, and the central contour was far away from the surrounding contours, indicating that the effect of the medium-to-flask ratio on the mycelial biomass was highly significant.

When the initial pH (B) and the medium-to-flask ratio (C) were constant, the effects of the incubation time (A) and the primary inoculum density (D) on the mycelial biomass (Figure 5c) and spore concentration (Figure 5i) of strain JMF-01 were investigated. The spore concentration of strain JMF-01 reached its maximum at an incubation time of 5.5–6.5 days, and the primary inoculum density was between 0.9 × 10^7^ and 1.5 × 10^7^ conidia/mL. The 3D surface plot showed a parabola with a large slope and an elliptical contour, indicating that the incubation time and primary inoculum density had a significant effect on the spore concentration. The contour of the mycelial biomass was nearly elliptical, indicating that the interaction between the incubation time and the primary inoculum density was significant.

When the incubation time (A) and the primary inoculum density (D) were constant, the effects of the initial pH (B) and the medium-to-flask ratio (C) on the mycelial biomass (Figure 5d) and spore concentration (Figure 5j) of strain JMF-01 were investigated. At a constant initial pH, mycelial biomass increased with increasing the medium-to-flask ratio, while spore concentration increased and then decreased. The slope of the response surface of mycelial biomass was large, while the slope of the surface of spore concentration was small, indicating that the effect of the medium-to-flask ratio on mycelial biomass was larger than that of spore concentration.

When the incubation time (A) and the medium-to-flask ratio (C) were constant, the effects of the initial pH (B) and primary inoculum density (D) on the mycelial biomass (Figure 5e) and spore concentration (Figure 5k) of strain JMF-01 were tested. At a constant primary inoculum density, the mycelial biomass and spore concentration both grew and then dropped with the rise in initial pH. However, the spore concentration increased with the increase in the inoculum level at a constant initial pH, indicating that the effect of the inoculation level on spore concentration was greater than that of the mycelial biomass. The contour plot was elliptical, indicating that the interaction between the initial pH and the primary inoculum density was more significant.

When the incubation time (A) and the initial pH (B) were constant, the effects of the medium-to-flask ratio (C) and the primary inoculum density (D) on the mycelial biomass (Figure 5f) and spore concentration (Figure 5l) of strain JMF-01 were investigated. The slope of the 3D surface plot of the spore concentration and mycelial biomass of strain JMF-01 was large, which indicated that the medium-to-flask ratio and primary inoculum density significantly affected the submerged culture growth of strain JMF-01.

### 3.4. Verification Test

The model calculated the optimal submerged culture conditions for the mycelial biomass; the incubation time was 5.68 days, the initial pH was 5.11, the medium-to-flask ratio was 0.43, and 1 mL of the primary inoculum with a spore density of 0.97 × 10^7^ conidia/mL was added, and the highest value of 0.35 g was obtained. The actual incubation time was 5.7 days, the initial pH was 5, the medium-to-flask ratio was 0.43, and the primary inoculum density was 1 × 10^7^ conidia/mL. The mycelial biomass was experimentally verified to be (0.29 ± 0.066) g.

The optimal submerged culture conditions for spore concentration were calculated by the model: the highest spore concentration of 6.49 × 10^8^ blastospores/mL was obtained when the incubation time was 6.32 days, the initial pH was 4.46, the medium-to-flask ratio was 0.35, and the primary inoculum density was 1.32 × 10^7^ conidia/mL of 1 mL. The actual incubation time was 6.3 days, the initial pH was 4.5, the medium-to-flask ratio was 0.35, and the primary inoculum density was 1.3 × 10^7^ conidia/mL. It was experimentally verified that the spore concentration was (5.88 ± 0.62) × 10^8^ blastospores/mL.

**Figure 6:**
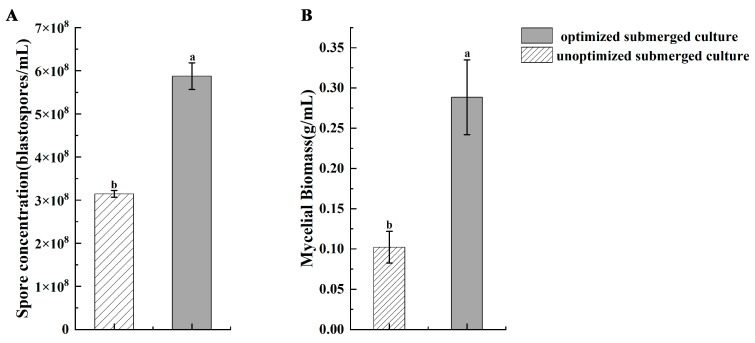
Validation of the statistical optimized submerged culture and unoptimized submerged culture. (**A**) Spore concentration. (**B**) Mycelial biomass. Data in the figure are the mean ± SEM. The SEM of three repetitions is shown in bars. The different lowercase letters (a, b) on the bars indicate a significant difference between different treatments (*p* < 0.05, ANOVA with Tukey HSD test).

Compared with the unoptimized medium conditions, the optimized submerged culture had the highest spore concentration (Figure 6A) and mycelial biomass (Figure 6B), which were 2.06 and 3.46 times higher, respectively. The validation tests were performed three times. The model’s ability to accurately represent the growth of strain JMF-01 under various submerged culture conditions can be seen by the small errors between experimental values and predicted values.

### 3.5. Pathogenicity Test

**Figure 7:**
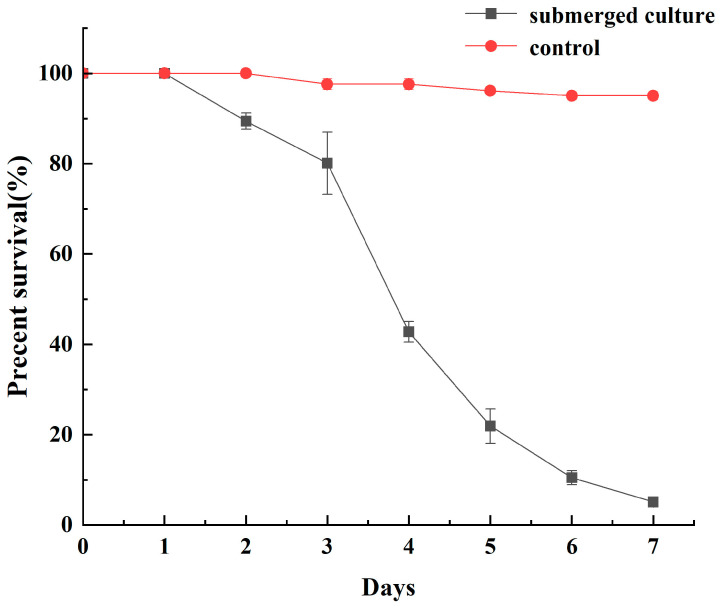
Survival of *M. persicae* treated with optimized submerged culture for 7 days. The control was sterile water. Submerged culture significantly decreased survival compared to control (χ^2^ = 25.816, df = 1, *p* < 0.001). The data in the figure are the mean ± SEM. The SEM of three repetitions is shown in bars.

Optimized submerged culture was obtained by using potato sucrose liquid medium (pH 4.5), the primary inoculum density was 1.3 × 10^7^ conidia/mL and medium-to-flask ratio was 0.35 (52.5 mL in each 150 mL flask) for 6.3 days. The survival of the optimized submerged culture after the treatment of peach aphids is shown in Figure 7.

## 4. Discussion

In this paper, the submerged medium suitable for the growth of strain JMF-01 was first selected, followed by single-factor tests to screen the optimal values of incubation time, primary inoculum density, medium-to-flask ratio, and initial pH. And then the interaction between the factors was evaluated using RSM to obtain a submerged culture process with a higher yield.

After incubation, the medium with the highest mycelial biomass and spore concentration produced by strain JMF-01 was potato sucrose liquid medium, and the optimal incubation time was 6–8 days. This result is similar to that of Pradeep et al. [57], who found that the mycelial dry weight and pigment yield of *Fusarium moniliforme* remained essentially constant after 8–10 days of incubation. The spore concentration of strain JMF-01 increased slowly after 6 days, while mycelial biomass showed a slight decrease after 6 days, probably due to the phenomenon of mycelial autolysis. The optimum incubation time in this study was similar to Tang et al. [58], who reported an optimum incubation time of 6 days for *Fusarium solani* in order to produce vitexin. Anellis et al. [59] found that six strains of *Clostridium botulinum* produced the maximum number of spores in 5–6 days, and Das et al. [60] incubated *Purpureocillium lilacinum* at 30 °C for 6 days to obtain the maximum number of spores. The creation of an essential medium that supported proper fungal culture growth and propagule formation was the first step in our optimization method. Depending on the nutritional needs for sporulation in submerged culture, the maximum spore concentration for some fungi can be a fixed value. Because of this, the development of incubation conditions can lead to more rapid sporulation. Therefore, reduced incubation time can often be the most important fungal biopesticide production method factor in reducing production costs.

The growth, development, and metabolism of fungi are all significantly influenced by a medium pH [53]. The results show that strain JMF-01 could grow over a wider medium pH range of 4.0–8.0. However, the maximum spore concentration and mycelial biomass of the strain were reached in the medium at pH 5, and the growth was lower outside of this medium pH range. The response surface optimization of the strain JMF-01 submerged culture spores and mycelia was optimal at an initial pH of 4.5 and 5.1, respectively, which is similar to the optimum pH for enzyme production of *Fusarium solani*, growth of *Fusarium oxysporum*, and maximum spore production of *Beauveria bassiana* [58,61,62,63]. However, the optimum pH for mycelial growth of *F. equiseti* found by Punja et al. [64] was 7.2–7.8. The capacity of entomopathogens to grow below pH 7 is beneficial during industrial production even if the pH of entomopathogens is ideal over a wide range because it allows for the reduction of substrate pH for the suppression of bacterial growth [65]. Moreover, different strains have different requirements for an initial pH.

Since DO is also likely to affect sporulation, these early flask culture observations may reflect changes in oxygen supply and demand brought on by biomass [66]. The cost of oxygen transfer is a significant part of the total production budget for mycelial cultures. The effect of aeration on the growth of strain JMF-01 was studied by changing the medium-to-flask ratio (volume of the medium to volume of the flask ratios (*v*/*v*)). The maximum biomass production and spore concentration were observed at a medium-to-flask ratio of 0.43 and 0.35 (*v*/*v*), respectively. According to Kumar et al. [67], *Aspergillus niger* showed the highest inulinase production at a medium-to-shake flask ratio of 1:20. In a study by Niaz et al. [68], *Aspergillus nidulans* recorded the highest extracellular lipase activity at a medium volume of 45 mL.

The inoculum size not only has a direct impact on the overall production yield, but it also affects the production cost [69,70,71]. We set the primary inoculum density to five values and screened the most suitable strain, JMF-01, by single-factor experiment to 1 × 10^7^ conidia/mL of spore suspension. The response surface optimization found that the optimum primary inoculum density for mycelium-producing biomass was 0.97 × 10^7^ conidia/mL, and the spore concentration was highest at 1.32 × 10^7^ conidia/mL. Our results were consistent with those of Santa et al. [72]. Alabdalall et al. [73] found that the optimum primary inoculum density for extracellular lipase production by *A. niger* was 2.5 × 10^7^ spores/mL. However, the mycelial biomass and spore concentration of strain JMF-01 decreased when the primary inoculum density was higher than 1 × 10^7^ conidia/mL or lower than 1 × 10^7^ conidia/mL. To balance the levels of nutritional components present in the submerged environment for ideal fungal development, the proper primary inoculum density is a crucial factor [73]. With a small inoculum size, it’s possible that there aren’t enough cells in the submerged culture to make use of the substrate needed to encourage the growth of fungal mycelium and the production of conidia. However, with a large inoculum size, the submerged medium may become more viscous due to the rapid growth of fungi, which could lead to a nutritional imbalance in the medium or excessive nutrient intake before the cells in the submerged culture are physiologically prepared to begin fungal production [34,74].

The beginning of sporulation is influenced by a wide range of environmental and nutritional conditions, and each species has unique characteristics. RSM is a well-established statistical technique that makes statistical predictions and evaluations while using economical experimental designs [75]. In this paper, BBD in RSM was applied to optimize the submerged culture conditions of strain JMF-01 to screen for optimal submerged culture conditions. The response surface methodology simplifies the experimental design into an optimization process of 29 trials, yet many factors and their interrelationships can be studied in less time with less labor.

The multiple coefficients of correlation, R and F values, were assessed in order to test the goodness of fit of the regression equation for strain JMF-01 submerged culture using the uniform design approach. The stronger the correlation between the obtained and predicted values, the closer R is to 1. Mycelial biomass and spore concentration both had R values of 0.9450 and 0.9355, respectively, indicating good agreement between experimental and predicted values. The F value is calculated as the ratio between the regression’s mean square and the real error’s mean square. In general, if the model accurately predicts the experimental data and the estimated factor effects are valid, the computed F value should be several times higher than the tabulated F value [76]. The estimated F values in this instance, which were 17.18 for the mycelial biomass and 14.49 for the spore concentration, exceeded the tabulated F values.

In this study, three-dimensional surface plots could clearly show that the medium-to-flask ratio significantly affected the mycelial biomass production of strain JMF-01, while the primary inoculum density and the initial pH significantly affected the spore production of the strain. The effect of medium and submerged culture conditions on the production of the entomopathogenic fungus, *F. equiseti*, has not been studied in depth. However, in other fungi, the positive induction of fungal production of spore and other metabolites by appropriate inoculum and an acidic initial pH has been documented. Santos et al. [77] reported that an acidic pH tends to increase the production of bikaverin by *F. oxysporum*. Chi et al. [78] found that a higher medium-to-flask ratio was more favorable for liquid culture aimed at obtaining mycelial biomass, and for liquid culture production in which spores have been obtained, the initial inoculum had a greater impact. According to Srivastava et al. [79], fungal growth appeared to be inversely correlated with pH variation since more fungal growth was observed when the pH was lowered.

## 5. Conclusions

The improvements in mycelial biomass and spore concentration that were attained through further optimization phases were around 3.46 and 2.06 times, respectively, larger than those attained under unoptimized conditions. Therefore, the optimization of submerged culture conditions had a great impact on the growth of strain JMF-01. Moreover, the optimized submerged culture was highly virulent against *M. persicae*, with a 95% mortality rate after 7 days (LT_50_ = 3.74 days). The results clearly support the previous conclusions of other researchers that optimized medium and submerged culture conditions are the main control for the entomopathogenic *F. equiseti*, as the main control factor for the production of biocontrol preparations [34,80].

## Figures and Tables

**Table 1:** Culture medium formula.

Number	Name	Components Per 1 L Distilled Water
1	Potato dextrose liquid medium	200 g peeled potato, 20 g glucose;
2	Potato sucrose liquid medium	200 g peeled potato, 20 g sucrose;
3	Sabouraud Dextrose Agar with yeast extract liquid medium	40 g glucose, 10 g peptone, 10 g yeast powder;
4	Czapek–Dox liquid medium	30 g sucrose, 3 g NaNO_3_, 0.5 g KCl, 1 g K_2_HPO_3_, 0.01 g FeSO_4_, 0.5 g MgSO_4_.7H_2_O;
5	Glucose yeast extract liquid medium	40 g glucose, 20 g yeast powder.

**Table 2:** The levels and code of variable chosen for BBD.

Factors	Levels		
	−1	0	1
A: Incubation time (d)	5	6	7
B: Initial pH	4	5	6
C: Medium-to-flask ratio	0.23	0.33	0.43
D: Primary inoculum density (10^7^ conidia/mL)	0.5	1	1.5

**Table 3:** BBD and the experimental results.

Experimental Serial No.	Incubation Time (d) A	Initial pH B	Medium-to-Flask Ratio C	Primary Inoculum Density (10^7^ Conidia/mL) D	Spore Concentration 10^8^ (Blastospores/mL)	Mycelial Biomass (g/mL)
1	6	4	0.33	0.5	4.55	0.24
2	6	6	0.43	1	3.41	0.35
3	6	5	0.33	1	6.01	0.31
4	7	5	0.23	1	4.58	0.12
5	7	6	0.33	1	3.22	0.26
6	7	4	0.33	1	5.35	0.22
7	6	5	0.33	1	5.84	0.28
8	6	5	0.33	1	5.98	0.29
9	6	5	0.33	1	5.86	0.31
10	6	4	0.33	1.5	6.01	0.24
11	6	5	0.43	1.5	5.27	0.32
12	5	4	0.33	1	4.50	0.29
13	6	5	0.23	1.5	6.06	0.18
14	6	4	0.43	1	5.63	0.34
15	5	6	0.33	1	4.14	0.26
16	6	6	0.23	1	4.41	0.13
17	6	5	0.43	0.5	4.55	0.32
18	6	6	0.33	0.5	3.18	0.24
19	6	5	0.23	0.5	4.38	0.14
20	7	5	0.33	1.5	5.58	0.26
21	5	5	0.33	1.5	4.58	0.27
22	6	6	0.33	1.5	4.31	0.26
23	5	5	0.33	0.5	3.52	0.26
24	5	5	0.43	1	4.13	0.35
25	7	5	0.43	1	5.13	0.28
26	6	5	0.33	1	6.63	0.33
27	5	5	0.23	1	4.60	0.19
28	6	4	0.23	1	4.52	0.16
29	7	5	0.33	0.5	2.57	0.28

**Table 4:** Analysis of variance of the spore production response surface model of the strain JMF-01.

Source	Sum of Squares	df	Mean Square	F-Value	*p*-Value	Significance
Model	27.04	14	1.93	14.49	<0.0001	significant
A	0.077	1	0.077	0.57	0.4611	
B	5.19	1	5.19	38.94	<0.0001	
C	0.016	1	0.016	0.12	0.7370	
D	6.83	1	6.83	51.21	<0.0001	
AB	0.79	1	0.79	5.91	0.0291	
AC	0.25	1	0.25	1.91	0.1889	
AD	0.96	1	0.96	7.19	0.0179	
BC	1.12	1	1.12	8.40	0.0117	
BD	0.028	1	0.028	0.21	0.6550	
CD	0.23	1	0.23	1.72	0.2105	
A^2^	7.17	1	7.17	53.79	<0.0001	
B^2^	5.08	1	5.08	38.09	<0.0001	
C^2^	1.34	1	1.34	10.05	0.0068	
D^2^	3.35	1	3.35	25.16	0.0002	
Residual	1.87	14	0.13			
Lack of Fit	1.45	10	0.15	1.39	0.4028	Not significant
Pure Error	0.42	4	0.10			
Cor Total	28.91	28				

Note: A, incubation time; B, initial pH; C, medium-to-flask ratio; D, primary inoculum density.

**Table 5:** Analysis of variance of the mycelial biomass response surface model of the strain JMF-01.

Source	Sum of Squares	df	Mean Square	F-Value	*p*-Value	Significance
Model	0.11	14	0.0078	17.18	<0.0001	significant
A	0.0037	1	0.0037	8.15	0.0127	
B	0	1	0	0.044	0.8374	
C	0.087	1	0.087	192.17	<0.0001	
D	0.0002	1	0.002	0.35	0.5635	
AB	0.0012	1	0.0012	2.72	0.1211	
AC	0.0000034	1	0.0000034	0.0076	0.9319	
AD	0.0001	1	0.0001	0.31	0.5862	
BC	0.0005	1	0.0005	1.07	0.3175	
BD	0.0002	1	0.0002	0.34	0.5707	
CD	0.0004	1	0.0004	0.80	0.3868	
A^2^	0.0032	1	0.0032	7.18	0.0179	
B^2^	0.0053	1	0.0053	11.80	0.0040	
C^2^	0.011	1	0.011	23.55	0.0003	
D^2^	0.0041	1	0.0041	9.02	0.0095	
Residual	0.0063	14	0.0005			
Lack of Fit	0.0051	10	0.0005	1.61	0.3428	Not significant
Pure Error	0.0013	4	0.0003			
Cor Total	0.12	28				

Note: A, incubation time; B, initial pH; C, medium-to-flask ratio; D, primary inoculum density.

## Data Availability

Not applicable.

## Supplementary Materials

The following supporting information can be downloaded at: https://www.mdpi.com/article/10.3390/microorganisms11010190/s1, Table S1: Single-factor test for the spore concentration by strain JMF-01 (mean ± SEM); Table S2: Single-factor test for the mycelial biomass by strain JMF-01 (mean ± SEM).
